# A synthetic microbial community for soybean biofertilization designed via chlorophyll-based iterative selection

**DOI:** 10.1128/aem.02548-25

**Published:** 2026-04-20

**Authors:** Damián Brignoli, Delfina Colla, Emilia Frickel-Critto, Cecilia B. Castells, Julieta Pérez-Giménez, Aníbal R. Lodeiro

**Affiliations:** 1 Instituto de Biotecnología y Biología Molecular (IBBM)-Facultad de Ciencias Exactas, Universidad Nacional de La Plata (UNLP) y CCT-La Plata, CONICET, La Plata, Argentina; 2 Cátedra de Genética-Facultad de Ciencias Agrarias y Forestales, Universidad Nacional de La Plata (UNLP), La Plata, Argentina; 3 Laboratorio de Investigación y Desarrollo de Métodos Analíticos, Universidad Nacional de La Plata (UNLP) y CIC-PBA, La Plata, Argentina; Danmarks Tekniske Universitet, Kgs. Lyngby, Denmark

**Keywords:** *Bradyrhizobium*, plant growth-promoting rhizobacteria (PGPR), nitrogen fixation, rhizosphere, microbiome

## Abstract

Improving the effectiveness of microbial inoculants for soybean is essential to enhance biological nitrogen fixation and reduce fertilizer dependence; however, inoculated *Bradyrhizobium* strains frequently display inconsistent field performance. Inoculation is usually carried out with single-strain formulations, overlooking the possible influence of the native soil microbiota on nodulation success. This limitation may be addressed by formulating inoculants with consortia that include selected members of the soil microbiota. To this end, a synthetic microbial community (SynCom) was developed through a host-mediated microbiome engineering approach guided by leaf chlorophyll content as a rapid, non-destructive plant trait. The experiment was initiated by inoculating soybean plants with a consortium of 9 *Bradyrhizobium* spp. and 14 non-rhizobial soil isolates. Across eight consecutive selection rounds under gnotobiotic conditions, rhizosphere communities associated with superior plant performance were pooled and propagated. Recurrent selection induced significant shifts in community composition, consistently favoring *Bradyrhizobium diazoefficiens* as the dominant nodulating member and enriching taxa from Pseudomonadales, Burkholderiales, and Sphingomonadales. Sequencing-based profiling and network analysis suggested the emergence of a cohesive and functionally enriched community, with increased potential for nitrogen transformations and organic matter turnover. When evaluated in non-sterile soil, the SynCom derived from the sixth selection round increased nodule number and biomass relative to an uninoculated control and a commercial inoculant strain. These results suggest that plant-guided selection can steer rhizosphere community assembly toward beneficial configurations and support the development of improved soybean bioinoculants.

**IMPORTANCE:**

Soybean [*Glycine max* (L.) Merr.] is a major global crop characterized by high seed protein content, which demands elevated nitrogen assimilation. To meet this demand, the crop can utilize atmospheric nitrogen through the process of biological nitrogen fixation in symbiosis with *Bradyrhizobium* bacteria, thus mitigating soil nitrogen depletion. Although *Bradyrhizobium*-based inoculants are applied at sowing, their interplay with other members of the rhizosphere microbiota remains poorly understood. It is well documented that plants and rhizosphere microbiota interact to shape plant growth and soil productivity. Therefore, this work evaluated the inoculation of soybean with a synthetic microbial consortium as a strategy to develop new-generation inoculants. These bioinputs are designed to harness plant-soil-microbe interactions to improve soybean productivity while preserving soil properties.

## INTRODUCTION

Nitrogen (N) is one of the most important nutrients in defining crop productivity. In legumes, N may be obtained from the atmosphere by symbiotic dinitrogen (N_2_) fixation in association with rhizobia, which reduce N_2_ to ammonia (NH_3_), thus making N available for plant nutrition. Particularly in soybean, a crop with global production exceeding 400 million tons across 146 million hectares in 2024/2025 (1), species from the *Bradyrhizobium* genus are mainly responsible for this beneficial interaction.

Soybean-nodulating *Bradyrhizobium* species originated in East Asia; however, once inoculated in new soils, these bacteria rapidly colonize the soil, generating allochthonous populations that can persist for a long time, competing against new inoculants for soybean nodulation (2–7). This complexity in soybean-nodulating *Bradyrhizobium* populations adds to that of soil microbiota, especially in the rhizosphere, the soil portion in close contact with roots. As a consequence of root activity, the rhizosphere differentiates from the surrounding soil as influenced by root respiration, pH modification, and exudation of diverse carbohydrates, amino acids, and organic acids, among other small organic molecules. These modifications of the soil environment give rise to niches heavily populated by a huge diversity of microorganisms—the so-called rhizosphere effect (8). Hence, inoculated *Bradyrhizobium* spp. strains must manage with their allochthonous relatives and the rhizosphere microbiota, both of which can either aid or counteract soybean root infection and nodulation. In addition, the plant is also populated by endophyte microorganisms that, when allocated in seeds, may be vertically transmitted through successive plant generations, influencing plant growth and development (9). Therefore, under the influence of endophytic and rhizosphere microbiota, root activity is modified and, in turn, the composition of these microbiota is modulated according to plant needs. Moreover, rhizosphere microbiota also modifies the properties of the surrounding soil. Hence, the plant-endophyte-rhizosphere microbiota-soil complex is currently considered a multi-organism or holobiont (10).

Important members of the rhizosphere populations are bacterial species that promote plant growth through various mechanisms, including the production of phytohormones, siderophores, phosphorus solubilization, and phytostimulation in addition to N_2_ fixation and therefore are known as plant growth-promoting rhizobacteria (PGPR) (11; reviewed in reference 12). In this context, some research highlights the benefits of co-inoculating rhizobia with other PGPR to enhance nodulation, N_2_ fixation, and other growth parameters, such as vegetative growth, yield, and biocontrol, instead of traditional inoculation, which relies on single-strains (13–20). However, research is needed to assess the extent to which inoculated *Bradyrhizobia*, alone or combined with PGPR, are able to invade soybean rhizosphere and modify its microbial composition, taking into account the possible resilience of these populations (21).

In natural environments, plant-associated microbial populations often co-evolve special interactions within these complex communities, which may play crucial agroecological roles (22–29). An emerging approach to harnessing these interactions is the artificial selection of microbial communities—also known as microbiome engineering or community-level selection (30)—giving rise to synthetic microbial communities (SynCom). Inspired by evolutionary principles, this methodology involves subjecting microbial communities to successive rounds of selection based on a desired phenotype in the host plant, such as leaf chlorophyll content or aerial biomass. Recent studies have demonstrated that this technique can modify both the structure and function of rhizosphere microbiomes, leading to reproducible phenotypic improvements in plants (31, 32). However, challenges remain, including microbial incompatibility, competition from native organisms, and adaptation to the target habitat.

In this study, an artificial selection strategy was implemented with the expectation of generating a SynCom with enhanced plant growth-promoting potential in soybean. The experiment started from a composite inoculant formed by 9 *Bradyrhizobium* spp. strains previously described (2) and 14 soil isolates with PGPR potential, which were subjected to a host-mediated microbiome engineering strategy by applying artificial selection over 8 plant-inoculation cycles using plant chlorophyll content as the selection criterion. It was hypothesized that recurrent selection guided by this measurable plant performance trait would shape the rhizosphere microbiota by favoring microbial taxa with superior symbiotic competence and growth-promoting capabilities. Chlorophyll content correlates well with plant N content and nodule biomass, both indicators of N₂ fixation capability (2). The resulting SynCom was then characterized both phenotypically and molecularly, and its capacity to promote plant growth and enhance *Bradyrhizobium*-mediated nodulation was evaluated under controlled conditions. In addition, the composition of rhizosphere microbiota was monitored at key steps of the selection procedure to evaluate the impact of initial application of the composite inoculant on rhizosphere diversity and resilience.

## RESULTS

### Recurrent inoculation strategy and SynCom selection procedure

To build up the SynCom, a recurrent selection strategy was employed, analogous to that previously applied to improve the motility of *Bradyrhizobium diazoefficiens* (33). In the present case, however, several additional challenges had to be addressed: the use of a complex bacterial consortium rather than a single parental strain, the indirect nature of the selection—based on the assumption that superior plant performance reflects a more effective microbiome—and the need to minimize the interval between successive selection steps to prevent destabilization of the consortium. Previous findings demonstrated that leaf chlorophyll content, measured non-destructively with a portable chlorophyllometer, correlates well with N₂-fixation parameters, such as total N content and nodule dry mass (2). Since chlorophyll readings provide an instantaneous measurement, they effectively overcome the time limitation and were therefore selected as the criterion for recurrent selection.

**Fig 1:**
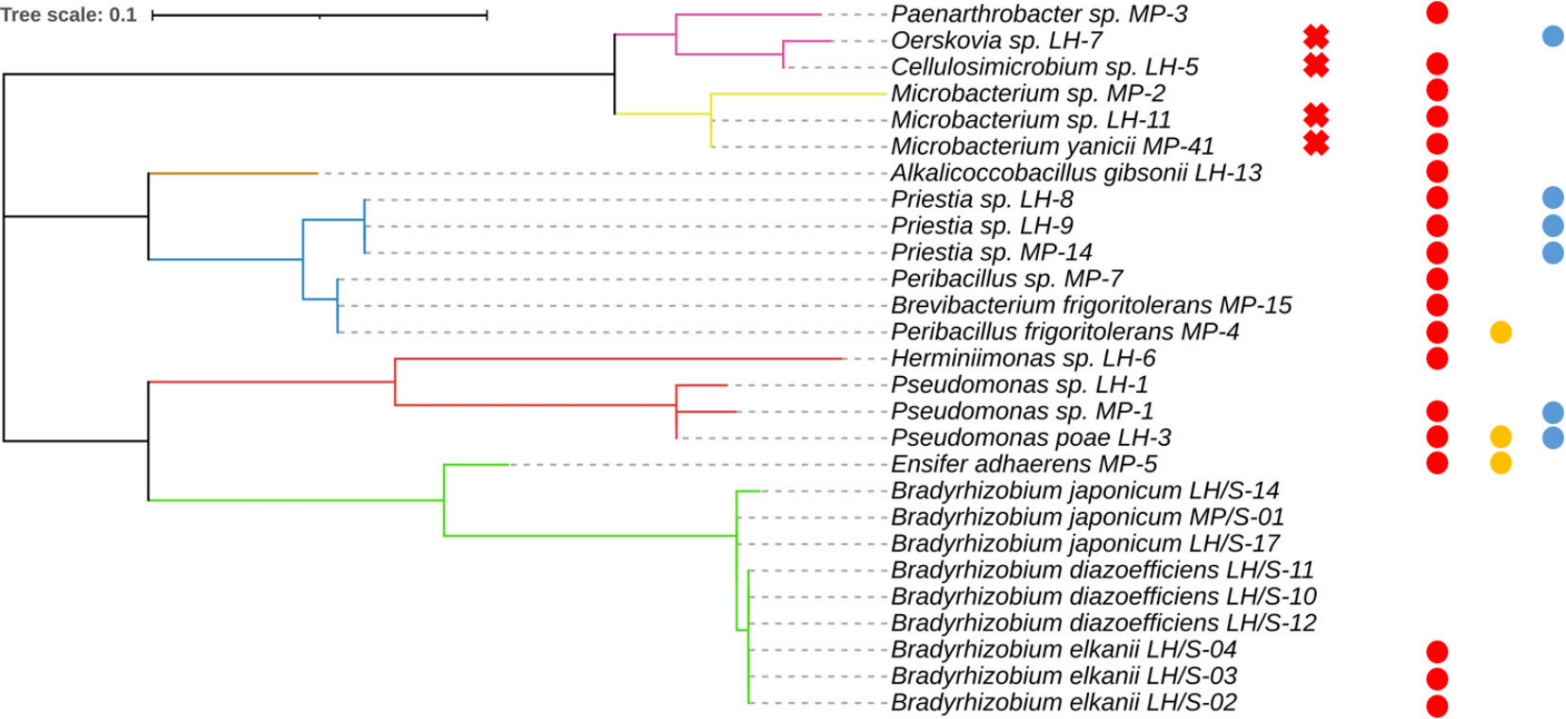
Maximum likelihood cladogram obtained from 16S rRNA gene (V1–V9) of soybean-nodulating strains and PGPR isolates. Branch lengths are shown to scale, indicating evolutionary distance used to infer the phylogenetic tree. Circles beside strain names indicate detected PGPR activities (red: IAA production, orange: siderophore production, light blue: phosphate solubilization). iTOL v7 was used for cladogram visualization. Red crosses indicate isolates that were discarded for further analyses due to their potential health risk.

At the beginning of the experiment, the SynCom was built up with 14 non-rhizobial strains belonging to 9 genera, most of which possessed PGPR properties (hereafter referred to as PGPR strains) plus 3 *B. diazoefficiens*, 3 *Bradyrhizobium elkanii*, and 3 *Bradyrhizobium japonicum* (Fig. 1). The PGPR strains were obtained from two different soils: six strains from a soil with previous history of soybean at Los Hornos (LH) and eight strains from a pristine soil without history of soybean at Mar del Plata (MP), both locations 370 km apart in the Province of Buenos Aires, Argentina (for detailed information, see reference 2). The rationale was to enrich the LH strains adapted to the soybean-cropped soil with MP strains that were presumed to be of high quality because of their adaptation to the pristine soil. Before use, strains with potential hazard (34) were removed from the collection (Fig. 1).

**Fig 2:**
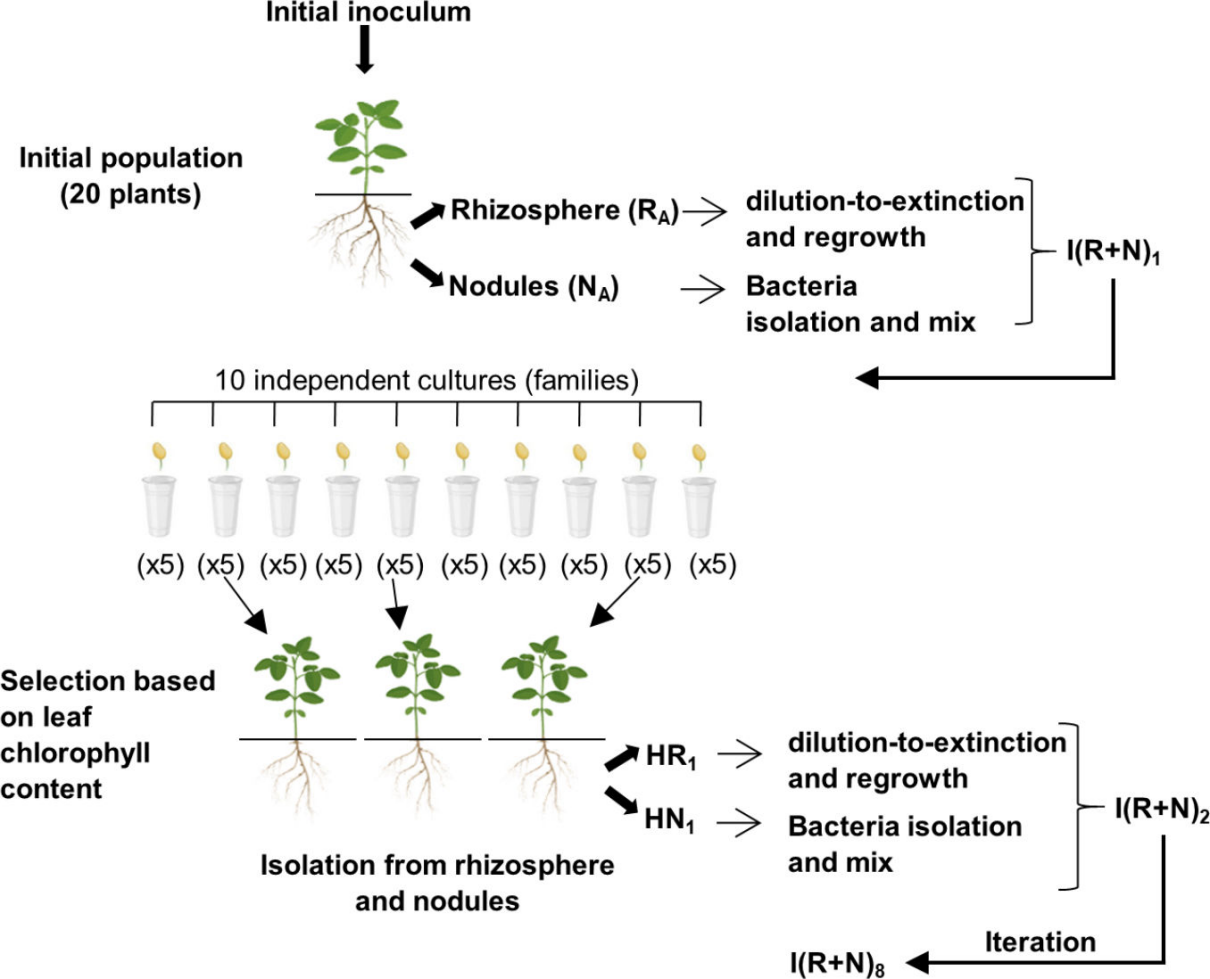
Experimental design for the artificial selection of soybean-associated microbial communities through the selection of plants with higher chlorophyll content. For acclimation of the bacterial mixture to the gnotobiotic environment of soybean rhizosphere, 20 soybean plants were inoculated with the initial mix of *Bradyrhizobium* spp. and PGPR. After 30 days in the greenhouse, the rhizosphere and nodules were recovered, and the mixture was prepared for the next selection round. Based on the similar growth rates of inoculated PGPRs, the rhizosphere microbiota was grown in G2MY, diluted to extinction to generate 10 replicas with different bacterial compositions, and regrown to obtain 10 cultures. In turn, bacteria from the nodules were mixed and added to each rhizosphere culture to stimulate competition for nodulation among the recovered rhizobia genotypes. Thus, the 10 independent cultures (families) were inoculated in groups of five plants each (50 plants in total). As controls, five plants were left uninoculated, and another five plants were inoculated with *B. japonicum* E109, the elite strain used in Argentina for industrial soybean inoculants. After 30 days in the greenhouse, the three plants with the highest leaf chlorophyll contents were selected, and their rhizospheres and nodules were processed as before, to generate the next round of selection. The entire process was repeated eight times. The diagram illustrates the flow of selection and propagation of the microbiome between rounds.

The whole procedure followed for this experiment is sketched in Fig. 2. Given that most of the PGPR strains chosen did not have previous contact with soybean plants or their endophytic bacteria, 20 soybean plants were inoculated with the mixture and cultivated in gnotobiotic conditions for 30 days (V2 phenological stage) to allow for microbial acclimation and selection within the soybean rhizosphere. After this period, rhizosphere and nodule bacteria were recovered separately. Rhizosphere bacteria, designated R_A_ (*R*hizosphere from *A*cclimation), were divided into two fractions: one was cultured in G2MY medium for reinoculation of plants, and the other was frozen for DNA extraction. On the other hand, rhizobia from nodules (N_A_, which stands for *N*odules from *A*cclimation step) were similarly divided and used for soybean reinoculation or frozen to obtain *Bradyrhizobium* DNA.

For the next steps, the generation of PGPR populations with different compositions was required, in order to enable the selection procedure among different groups of plants. Since the species composition of rhizosphere communities cannot be determined in a time lapse sufficiently short to avoid changes in population stability and composition before reinoculating the next series of plants, cultures were diluted to extinction to favor stochastic variation in composition (35). Therefore, bacteria collected from rhizospheres, starting from R_A_, were inoculated together in 10-mL G2MY, grown, and diluted in 10 replicas in order to inoculate 10 G2MY flasks with ~50–100 bacteria each. After growing again, each culture was used to inoculate a set of five plants. In this way, it was expected that each plant set received a PGPR community with different strain compositions. The same procedure was followed for the next rounds of selection (Fig. 2).

Regarding *Bradyrhizobium* spp., the objective was to generate both a competition for nodulation and an adaptation to PGPRs among the strains recovered from nodules at the previous selection round. Since nodules provide a high number of rhizobia whose physiological state is already optimized for their interaction with soybean plants, bacteria extracted from N_A_ were mixed, divided into 10 aliquots, and added directly to each one of the 10 PGPR rhizosphere broths, constituting the first inoculum for the next selection round (Fig. 2).

Therefore, 10 parallel samples were generated, each one with—presumably—a distinct PGPR composition and all the *Bradyrhizobium* spp. obtained from the nodules of the previous round. These 10 samples were designated I(R+N)_1._*_j_* (where *j* = 1–10), representing the inoculated mixture of rhizosphere plus nodule bacteria for the 1st round and its *j*th replica. The 10 five-plant sets inoculated with [I(R+N)_1.1_ to I(R+N)_1.10_] constituted 10 selection “families.” Control and reference groups were inoculated with sterile G2MY or *B. japonicum* E109, which is the reference strain recommended by INTA for soybean in Argentina (36). After 30 days in the greenhouse, the three plants from different families with the highest leaf chlorophyll contents (measured as SPAD values) (37) were selected and harvested rhizosphere (HR_1_) and harvested nodule (HN_1_) bacteria from these plants were recovered as for the acclimation step and pooled to initiate the second round. This entire procedure was iterated eight times, from April to December 2024.

**Fig 3:**
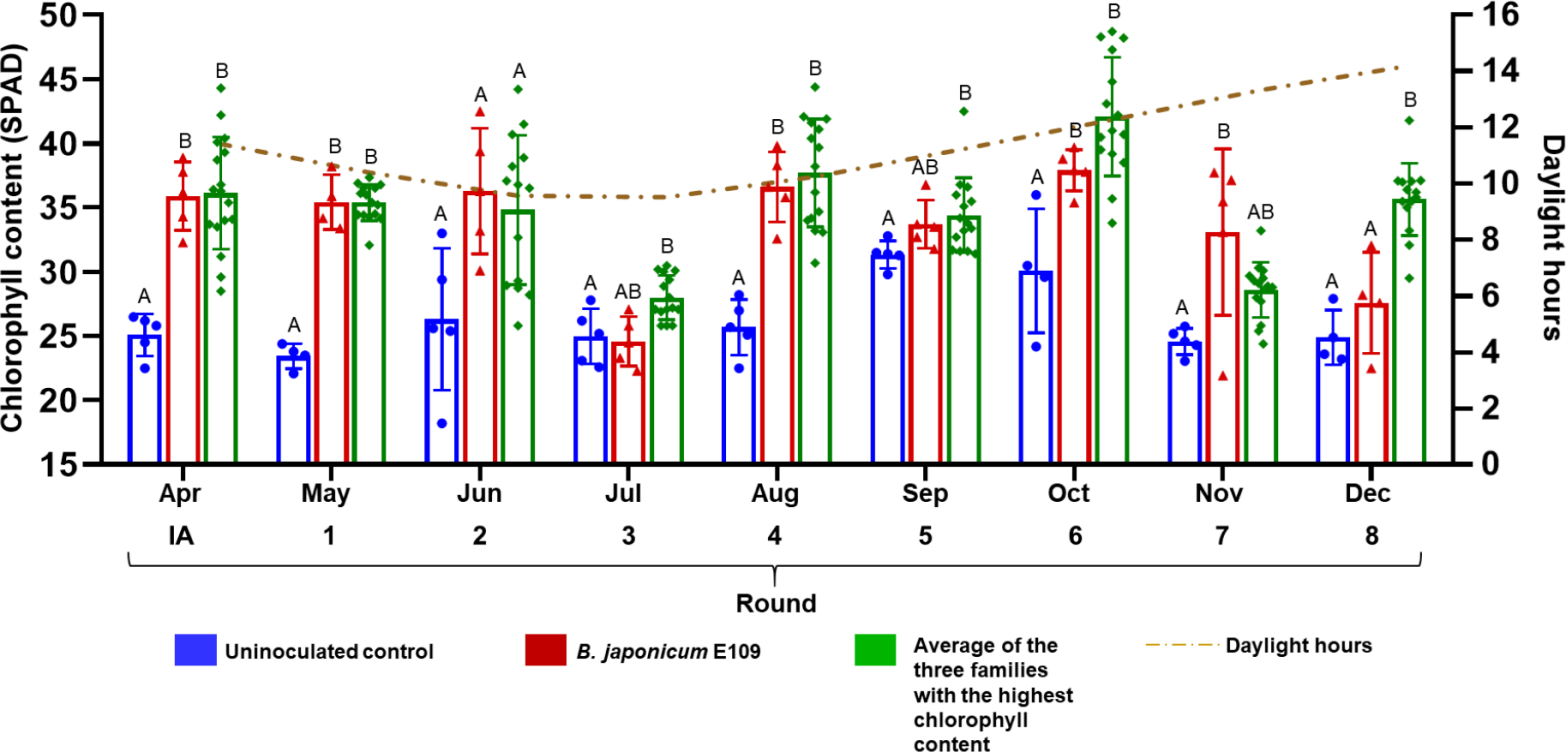
Chlorophyll contents (SPAD units) in leaves of soybean plants subjected to eight rounds of recurrent selection for rhizospheric microbiome enhancement. Bars represent averages ± SD and letters on the bars indicate the statistical difference as determined by one-way analysis of variance, followed by Tukey’s test (α = 0.05); columns with the same letter are not significantly different. The green bars represent the mean chlorophyll content of the plant families to which the top-three plants, according to their SPAD values, belonged. Dots indicate individual plant values. The rhizospheres and nodules from the top three plants were pooled at 30 days after inoculation and reinoculated for a new selection round. Blue and red bars represent uninoculated controls and *B. japonicum* E109-inoculated reference plants, respectively, each based on five plants per cycle. The green dashed line indicates the quantity of daylight hours along the period.

Throughout the experiment, SPAD values tended to be lower in uninoculated controls than in plants inoculated with the reference strain *B. japonicum* E109. In addition, the SynCom performed similarly to E109 during the initial acclimation and the first two selection rounds, indicating that, at minimum, the SynCom matched the effectiveness of the recommended elite strain. From selection round 3 onward, however, SPAD values of selected SynCom-inoculated plant families tended to be higher than those of E109-inoculated plants (with the exception of round 7 which, for unknown reasons, rendered lower SPAD values), reaching statistical significance in round 8 (Fig. 3). The highest SPAD values were recorded in round 6 in the SynCom family, although the difference with E109 was not statistically significant in this round. Variations in chlorophyll content among rounds may be caused by differences in solar radiation, because, although the experiment was conducted in the greenhouse under controlled conditions and artificial light, it also received sunlight. Nevertheless, graphical analysis showed no correlation between chlorophyll levels and natural daylight hours.

The best performance of the SynCom was obtained in rounds 6 and 8 (Fig. 3). Therefore, to compare the microbial composition in these rounds with the initial one, DNA samples from N_A_, HN_6_, R_A_, HR_6_, and HR_8_ were selected for further analysis. In addition, DNA from rhizosphere bacteria of uninoculated controls was prepared, constituting the R_0_ sample.

### Bradyrhizobial genotypes along nodules passage during recurrent selection

To assess rhizobial genotypic diversity, BOX-A1R DNA fingerprinting was performed (38). This method was selected for its speed and sensitivity in differentiating strains, despite its known susceptibility to minor inter-experimental electrophoretic profile variations. Nodules were randomly chosen at the acclimation and sixth round steps, rhizobia were isolated from them, and DNA fingerprints were analyzed. In the N_A_ sample, isolates formed a distinct cluster related to *B. diazoefficiens* reference strains, suggesting *B. diazoefficiens* early establishment as a major nodulating partner (see Fig. S1A at http://sedici.unlp.edu.ar/handle/10915/191256). By contrast, in the HN_6_ sample, collected 7 months after the initial inoculation, two distinct clades emerged (see Fig. S1B at http://sedici.unlp.edu.ar/handle/10915/191256). One clade grouped closely with the inoculated *B. diazoefficiens* strains while the other, although still similar to *B. diazoefficiens*, was less related to the inoculated strains.

The affiliation of all these isolates with *B. diazoefficiens* was further supported by the presence of the *nosZ* gene in all of them. This gene, encoding nitrous oxide reductase, is present in all three *B. diazoefficiens* strains used in this study but is undetectable in the six *B. elkanii* and *B. japonicum* strains (2). Taken together, these results suggest that selective pressures during recurrent selection of plants with high leaf chlorophyll contents favored *B. diazoefficiens* genotypes within the nodulating population.

### Microbial community dynamics during SynCom selection

The V3–V4 DNA regions of the 16S rRNA gene from pooled rhizosphere samples were sequenced. Following sequence quality control and cleaning, amplicons with approximately 450 bp in size were obtained and aligned to the SILVA database for taxonomic assignment. The number of base pairs and reads obtained for each round is summarized in Table S1 at http://sedici.unlp.edu.ar/handle/10915/191256.

**Fig 4:**
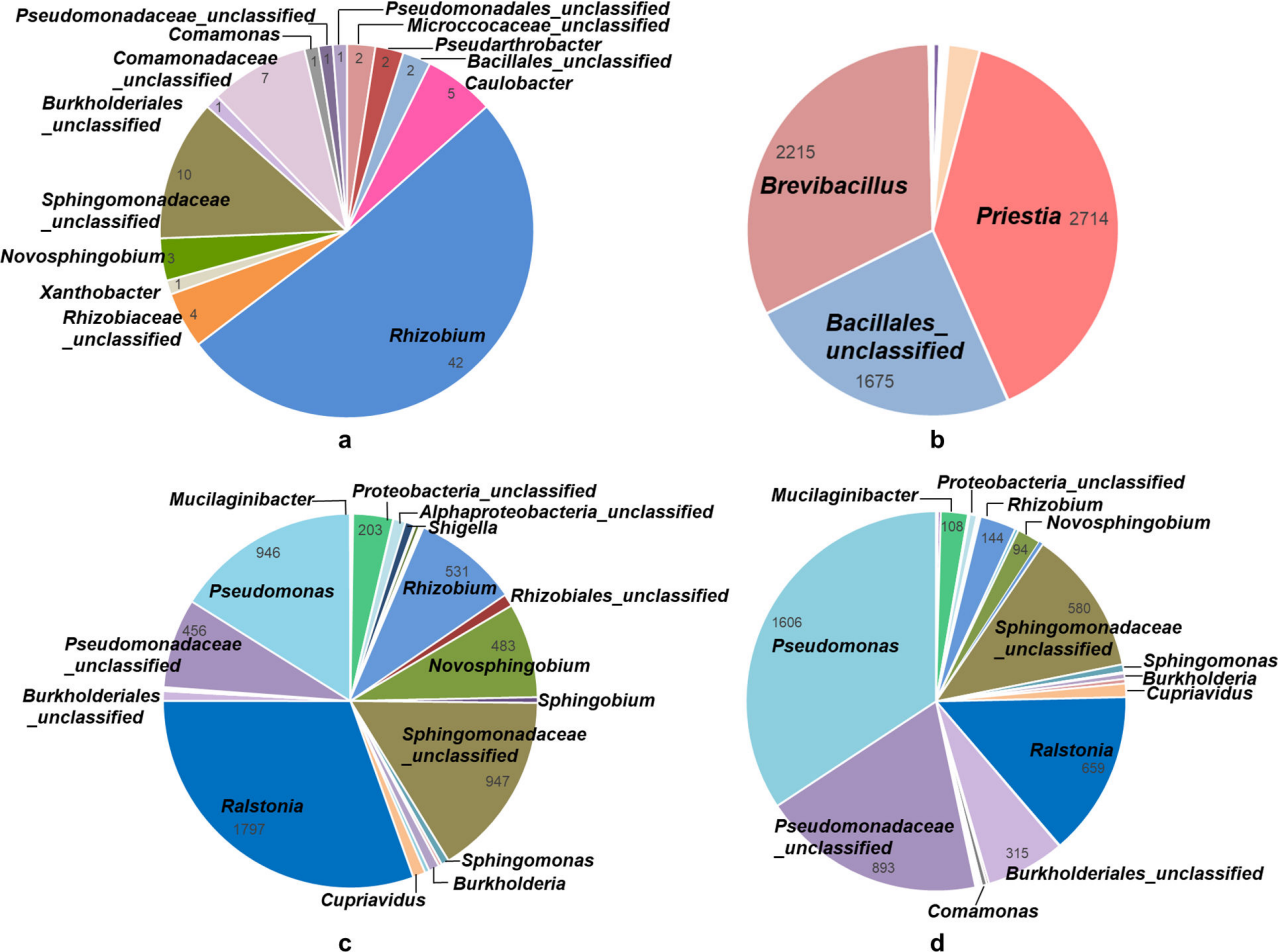
Temporal shifts in rhizospheric microbial composition across selection and inoculation cycles. (**a**) Uninoculated plants (R_0_); (**b**) R_A_; (**c**) HR_6_; (**d**) HR_8_. OTUs were identified by 16S rRNA gene (V3–V4) amplicon sequencing. Pie charts show the relative abundance of the most prevalent bacterial taxa classified at the genus or lowest assigned taxonomic level in each pooled sample. Colors are consistently assigned across cycles to facilitate comparison. Numerical values indicate the number of OTUs assigned to each genus, highlighting changes in community structure throughout the artificial selection process.

Various bacterial taxa were detected in the uninoculated R_0_ sample (Fig. 4a). These microorganisms likely originated from two sources: seed endophytes that were not completely removed during seed surface sterilization and environmental bacteria that entered the pots, which remained open in the greenhouse throughout the experiment. Such endophytic or environmental taxa were expected to also colonize the rhizospheres of inoculated plants. A considerable proportion of *Rhizobium* operational taxonomic units (OTUs) was detected in uninoculated controls; however, none of these plants formed nodules, suggesting that these microorganisms were not effective soybean symbionts. These strains might be of endophytic origin, but they could also be environmental in nature, as other research groups working with *Rhizobium* and *Sinorhizobium* also operate in the same greenhouse.

**Fig 5:**
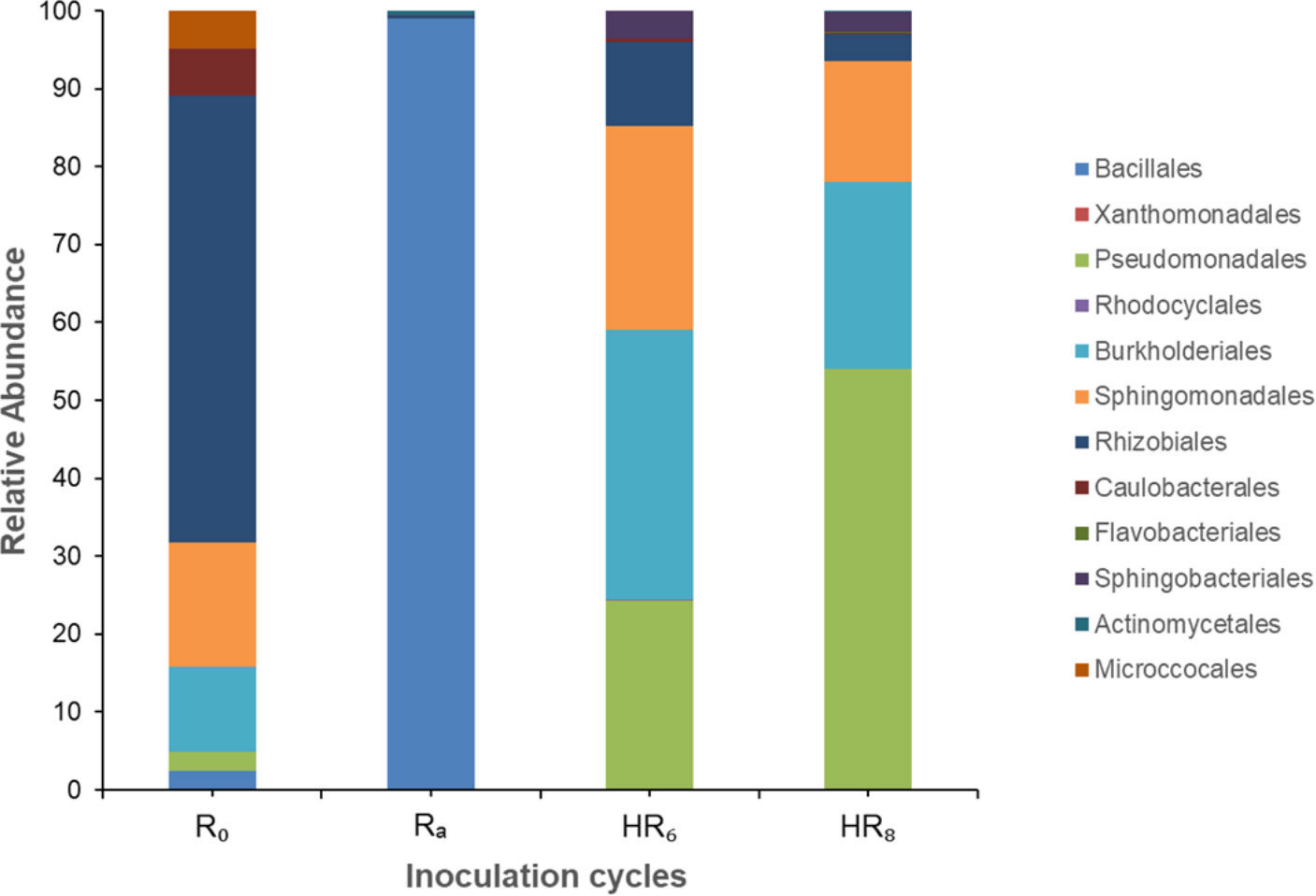
Relative abundance (%) of the main bacterial orders along the recurrent selection process.

After inoculation with the multi-species broth, *Bacillus*-related taxa became overwhelmingly dominant in R_A_, reflecting massive colonization by this kind of bacteria (Fig. 4b). Subsequently, the community structure progressively shifted back toward the initial pattern observed in non-inoculated plants, as seen in HR_6_ (Fig. 4c) and HR_8_ (Fig. 4d). This reestablishment was accompanied by a marked decline in the proportion of *Rhizobiales* and their gradual replacement by *Pseudomonadales*, *Burkholderiales*, and *Sphingomonadales*, which were initially present at low abundance but increased over successive rounds, particularly *Pseudomonadales* (Fig. 5). The reduction in *Rhizobiales* may be related to the invasion of *Bradyrhizobium* spp. after nodulation and reinoculation cycles, potentially suppressing other members of the order. Thus, the observed restoration of the baseline community structure—except for the increased representation of *Pseudomonadales*, *Burkholderiales*, and *Sphingomonadales* at the expense of *Rhizobiales*—suggests a degree of resilience within the system, likely driven by seed endophytes and associated bacterial groups. At the same time, the recurrent selection process appears to promote a community enriched in taxa with potential plant-beneficial traits. Nonetheless, these findings underscore the difficulty of maintaining a desired PGPR population in the mid- to long-term, across harvest and re-sowing cycles.

Dynamic changes in microbial composition were assessed throughout the experiment using species richness and Shannon diversity as indicators of community structure. The Shannon diversity index (H) increased after inoculation (from 2.47 in R_0_ to 4.43 in R_A_) and then exhibited a progressive decline throughout the experiment, indicating a reduction in both evenness and overall diversity (from 4.43 in R_A_ to 4.17 in HR_6_ and 3.51 in HR_8_). This pattern suggests a gradual simplification of the rhizospheric microbiota as a consequence of the selection process. The observed changes in the rhizosphere microbial community may also be appreciated in a non-metric multidimensional scaling (NMDS) analysis (see Fig. S2 at http://sedici.unlp.edu.ar/handle/10915/191256). The R_0_ sample was located in a region clearly separated from the plane, suggesting that its microbial community differed markedly from those associated with inoculated plants. After the initial inoculation, the microbial composition in R_A_ shifted substantially, reflecting a community transition resulting from the invasion of the ecosystem by the inoculant. In turn, samples HR_6_ and HR_8_ clustered closely together, again suggesting high similarity between these communities and a certain resilience of the system. This spatial arrangement in the NMDS suggests a directional trajectory: an abrupt change after the initial inoculation, followed by a progressive community stabilization between the intermediate and final rounds. This pattern is also reflected in the Bray-Curtis dissimilarity matrix (see Table S2 at http://sedici.unlp.edu.ar/handle/10915/191256), which shows a smaller distance between the last rounds analyzed. To further explore differences between communities, exploratory composition analyses, including SIMPER, were applied. In this context, the analysis identified the genera that contributed most to the differences observed between communities throughout the selection process. Table S3 at http://sedici.unlp.edu.ar/handle/10915/191256 indicates that, within this exploratory framework, the genera *Pseudomonas* and *Ralstonia* were the ones that contributed most to the differences observed between rounds.

To gain insights into the potential ecological roles of the rhizosphere microbial communities, functional inference was performed using the FAPROTAX database (see Fig. S3). This analysis mapped taxonomic affiliations of OTUs to putative metabolic functions based on curated literature associations. The most prevalent predicted functions included organic matter degradation and various N cycling pathways, reflecting the key roles of these communities in nutrient turnover. Additional enriched functions encompassed sporulation, antibiotic biosynthesis, and potential pathogenicity or mutualism, indicating the presence of taxa involved in stress resilience and microbial interactions. In contrast, functions such as hydrocarbon degradation, bioremediation, and resistance to environmental stressors appeared less represented, suggesting limited occurrence of taxa specialized in these capacities under the conditions tested.

Finally, co-occurrence analyses were conducted considering either all selection rounds or only the final rounds (HR_6_ and HR_8_). When all rounds were included, the resulting network showed no clear modular structure, with the initial dominance of *Rhizobium* being suppressed and integrated into a more diverse and cohesive community (see Fig. S4A at http://sedici.unlp.edu.ar/handle/10915/191256). Application of the Louvain algorithm (39) revealed a low modularity value (Q = 0.059). Together with the high clustering coefficient (0.926), this result indicates that successive rounds of selection promoted the development of a microbial consortium characterized by complex and widespread interactions, rather than discrete, isolated modules. Thus, the community emerging from serial passages appears highly interconnected, forming a cohesive network in which local subgroups remain strongly linked.

Analysis restricted to HR_6_ and HR_8_ yielded similar results (see Fig. S4B at http://sedici.unlp.edu.ar/handle/10915/191256), with an even higher clustering coefficient (0.98) and a very low modularity value (Q = 0.005). In this network, the mean degree of connectivity was 24.5, indicating that, on average, each node was directly associated with 24–25 other microorganisms. This reflects a highly dense network functioning as a single, unified community. These findings suggest that the selective pressure applied during recurrent selection fostered the emergence of a system in which widespread cooperation among microbial members predominates.

### Performance of selected SynCom in non-sterile soil

The SynComs HR_6_ and HR_8_ were evaluated in pots filled with non-sterile soil from the UNLP experimental field at Los Hornos, the site from which most of the *Bradyrhizobium* spp. and 6 out of the 14 PGPR strains used in this study were originally isolated, to see whether the performance of bradyrhizobia may be enhanced by the PGPR community. Uninoculated pots were used as controls, and pots inoculated with the elite strain *B. japonicum* E109, as reference. As shown in Table 1, no significant differences were observed between control and reference treatments, confirming the presence of effective soybean-nodulating rhizobia in this soil. In contrast, the performance of the SynComs differed markedly. While HR_8_ behaved similarly to the control and reference treatments, HR_6_ promoted higher nodule number, nodule dry weight, and shoot dry weight. Moreover, principal component analysis clearly separated HR_6_ from the other treatments, with its position driven by the above-mentioned traits (see Fig. S5 at http://sedici.unlp.edu.ar/handle/10915/191256). Unlike the results obtained in the recurrent selection rounds, chlorophyll content did not differ among treatments, likely reflecting the better nutritional status of the soil compared with the vermiculite substrate used at selection.

**TABLE 1:** Symbiotic performance of SynComs (HR_6_+HN_6_) and (HR_8_+HN_8_) in comparison with *B. japonicum* E109 as reference strain or uninoculated control in non-sterile soil from the UNLP experimental field at Los Hornos, Argentina^*a*^

Treatment	Nodules plant^−1^	Dry wt (mg)				Shoot/root ratio	Chlorophyll content (SPAD units)
		Total nodules	Individual nodules	Shoot	Root		
(HR_6_+HN_6_)	24 b	64.2 c	2.7 d	775 c	665 b	1.4	33.3 a
(HR_8_+HN_8_)	18 b	31.4 abc	1.8 abc	596 abc	364 ab	1.7	33.3 a
*B. japonicum* E109	15 ab	24.5 abc	1.7 abc	700 bc	512 b	1.4	33.6 a
Uninoculated control	18 b	34.1 bc	1.9 bc	670 bc	522 b	1.3	35.0 a

^*a*^ Different letters indicate significant differences (*P *< 0.05).

## DISCUSSION

The integral role of the microbiota in plant fitness is widely recognized and central to the plant holobiont concept (40). Understanding microbial community evolution under artificial selection provides a strategy to enhance plant–microbe interactions, which may be harnessed for sustainable agriculture. Several studies have demonstrated the capacity to improve rhizospheric microbial consortia by using a plant trait for selection, including plant biomass, flowering time, resistance to pathogens or herbivory, among others (41). In this context, recurrent selection to obtain a microbiome for enhanced N_2_-fixation in soybean was addressed here. To this end, the experiment was conducted under hydroponic, axenic conditions by selecting subpopulations of the rhizosphere community and replacing them in each selection round. Therefore, the aim was not to study the adaptation of a given community to the soybean rhizosphere, but to select a synthetic community (SynCom) able to enhance the N_2_-fixing performance of inoculated *Bradyrhizobium* spp. strains. However, commonly used plant phenotypes, such as shoot or nodule dry weight or total plant N content, were not suitable as selection criteria. These measurements are not only destructive but also require extended periods to complete, exceeding the time during which the selected microbiome remains physiologically stable and compositionally consistent before its transfer to the next inoculation round. Alternatively, direct N_2_-fixation estimates using the acetylene reduction assay (ARA) or measuring hydrogen (H_2_) evolution might have been used. However, these approaches were not feasible in our case. Moreover, ARA is known to induce a rapid decline in nitrogenase activity, leading to inaccurate estimates (42), while H_2_ evolution measurements require *Bradyrhizobium* strains incapable of recycling H_2_ for the generation of extra ATP (43), a trait that has not been characterized in the strains used in this study. Therefore, chlorophyll content in leaves was used as the selection criterion since this trait correlates well with N_2_-fixation parameters (2) and can be measured instantaneously and non-destructively using a portable chlorophyllometer, thereby providing a practical and reliable approach for guiding recurrent selection.

The SynComs obtained after eight rounds of selection seemed stable. In particular, the SynCom HR_6_+HN_6_ impacted important N_2_-fixing parameters, such as nodule number and dry weight in non-sterile soil, a promising result taking into account that the comparison was made against an elite strain (36), in a bradyrhizobia-populated soil. Molecular characterization demonstrated dynamic restructuring of the microbial community, with *B. diazoefficiens* genotypes dominating within nodules, accompanied by the emergence of a highly interconnected and functionally cohesive rhizospheric consortium.

Dominance of *B. diazoefficiens* in nodules population may be explained in part by the selection criterion used, among the nine *Bradyrhizobium* spp. strains employed, two of the three *B. diazoefficiens* promote the highest chlorophyll contents when inoculated alone (2). However, this interpretation does not apply to the N_A_ population, where no selection was still applied and cannot explain the rise of two clades in HN_6_. Therefore, it seems that other selection pressures took place in the rhizosphere or during competition for nodulation. These results are also in line with previous observations of allochthonous soybean-nodulating populations, whose DNA fingerprints diverge from those of collection strains, in particular those used in commercial inoculants (2, 3, 44–46). Thus, unknown selection pressures seemed to shape the genotypic structure of the soybean-nodulating bradyrhizobial population at a rate faster than previously estimated.

The SynCom selection process, conducted in sterile vermiculite, resulted in a dynamic assembly of the rhizosphere microbiome with a core microbiome, including *Pseudomonas*, *Rhizobium*, and members of the *Sphingomonadaceae* family. Of note, the dominance of *Bacillales* in the R_A_ community seems to have been suppressed later by serial passages. *Pseudomonas* exhibited stable persistence across all cycles, *Rhizobium* dominated in the uninoculated controls, and *Sphingomonadaceae* members were consistently present. These observations align with previous findings identifying *Pseudomonas* as a dominant genus in soybean (47). The presence of various genera commonly associated with seeds, such as *Pseudomonas*, *Pantoea*, *Methylobacterium*, *Bacillus*, *Sphingomonas*, *Curtobacterium*, and *Microbacterium* (48–51), suggests that seed microbiota, in addition to horizontal acquisition from the environment (52), played a role in shaping the rhizosphere community, potentially exerting priority effects (53).

Functional inference using the FAPROTAX database indicated an enrichment of microbial pathways related to organic matter degradation and N cycling, crucial for nutrient availability in soil (54–56). Other predicted functions included sporulation, antibiotic production, and host–pathogen interactions, suggesting potential mechanisms for microbial resilience and interaction within the SynCom. Co-occurrence network analysis revealed that the SynCom obtained is organized as a “small-world” network. It is characterized by a single, massive core of microorganisms (explained by the very high clustering coefficient) that cannot be divided into meaningful sub-communities (explained by the very low modularity), along with some peripheral members. This result was obtained both if all microbiomes were included or if only HR_6_ and HR_8_ were considered.

In a closed system with limited resources (such as the rhizosphere of plants growing in a pot of vermiculite with mineral solution, as in the present case), microbes that may thrive over the long term should be able to develop interdependencies, such as cross-feeding or co-metabolism, thus creating positive connections as edges in the network. Therefore, we may interpret that a group of microbes that, as a whole, is more efficient at utilizing rhizosphere resources will have more biomass and will be better transferred to the next passage, favoring integration over modularity. These facts might also explain the integration of the rhizospheric consortium with the *B. diazoefficiens* nodule occupants.

Beyond suggesting the feasibility of chlorophyll-guided microbiome engineering, our findings have practical implications for the design of microbial bioinoculants. The initial SynCom deliberately combined strains originating from a soybean-cultivated soil with others isolated from a pristine soil lacking soybean history, thereby integrating microorganisms with contrasting ecological backgrounds. The recurrent selection was conducted in a simplified, gnotobiotic system in which community restructuring was driven primarily by plant-mediated filtering and interactions among inoculated strains, together with contributions from seed endophytes and incidental environmental microorganisms. However, when transferred to non-sterile soil containing established rhizosphere and soybean-nodulating populations, the performance of the selected communities diverged, with HR_6_+HN_6_ enhancing nodulation traits, whereas HR_8_+HN_8_ did not. This contrast indicates that SynCom efficacy might depend on its capacity to integrate into pre-existing soil microbiomes. Consequently, the prospect of a universally effective soybean consortium appears limited. Rather than seeking a broadly applicable microbial mixture, our results support the development of synthetic communities that are assembled from diverse functional pools but subsequently validated and refined under representative soil conditions. The degree of specificity required may correspond to agroecological regions with comparable soil properties and resident microbial assemblages, rather than individual fields. In this sense, plant-guided iterative selection provides a tractable framework for generating context-adapted bioinoculants capable of modulating, rather than overriding, native soil microbial networks.

## MATERIALS AND METHODS

### Bacterial strains and inoculant preparation

The *Bradyrhizobium* spp. strains used in this study were previously described (2). These strains, as well as PGPR strains, were obtained from a soybean-cultured soil at Los Hornos (LH), Province of Buenos Aires, Argentina (34°59′10.5″ S; 58°59′58.9″ W), and from a pristine soil located at Mar del Plata (MP), Province of Buenos Aires, Argentina (38°4′23.0″ S; 57°33′34.5″ W). The properties of these soils were described elsewhere (2).

Non-rhizobial bacteria were extracted from soils as described (57). Briefly, soil (5 g dry mass equivalent) was suspended in 50 mL sterile water and shaken at 180 rpm for 1 h. The mixture was then sonicated twice with an ultrasonic bath (47 kHz, 1 min per immersion) before a final 30 min shaking step. The suspensions were filtered through a 50 μm filter and then through a 10 µm filter. To obtain culturable isolates for plant inoculation, serial dilutions were performed, and a 100 µL aliquot was seeded in Petri dishes with YMA (58), TSA (Difco Laboratories, Detroit, MI, USA), and SDA (Oxoid, Dardilly, France) and incubated at 28°C and 37°C. Isolates were maintained as glycerol stocks as described (3).

Isolates were evaluated for known PGPR properties in plate assays for phosphate solubilization in NBRIP agar (59) and siderophore production in CAS agar (60). In addition, IAA production was evaluated in G2MY broth supernatants by high-performance liquid chromatography using purified IAA (Sigma-Aldrich, Buenos Aires, Argentina) as a standard, according to Jensen et al. (61).

To assemble the initial inoculum for SynCom development, 18 rhizosphere isolates encompassing a range of divergent colony morphologies and sizes were chosen and classified in 11 genera (Fig. 1; also see Fig. S6 at http://sedici.unlp.edu.ar/handle/10915/191256), discarding those identified as *Cellulosimicrobium* sp., *Oerskovia* sp., *Microbacterium yanicii,* and *Microbacterium* sp. because of their potential hazard after consulting the list of microorganisms recommended under the qualified presumption of safety by the EFSA Panel on Biological Hazards (34). The chosen isolates, as well as *Bradyrhizobium* spp., were grown in G2MY medium that contained (in g L^−1^ distilled water): glucose, 5; sodium gluconate, 5; yeast extract, 2.2; mannitol, 5; CaCl_2_, 0.9; MgCl_2_, 0.3; K_2_HPO_4_, 1.1; and KH_2_PO_4_, 0.9 (2). Growth was monitored by spectrophotometer readings of optical density at λ = 500 nm (OD_500_) and CFU counts. All rhizosphere isolates grew in G2MY culture medium with similar doubling time (~2.5 h), while the slow-growing *Bradyrhizobium* strains had a doubling time of ~9.0 h in this medium. Taking into account that the present development is intended for a new inoculant, growth conditions were simplified for plant experiments by preparing two separate broths in G2MY: one with the rhizosphere isolates and another with the *Bradyrhizobium* spp. These two cultures were grown separately until each one attained OD_500_ ~0.5 and mixed at the moment of inoculating soybean plants.

### Plant experiments

Soybean seeds (Bioceres 4.12 RR) were surface-sterilized and germinated as described (2). After germination, 20 plantlets with 3 cm-long roots were sown in 500-mL plastic pots filled with vermiculite (one plant per pot) and watered with 200 mL N-free modified Fåhraeus plant nutrient solution (MFS) (62).

Since most of the rhizosphere isolates were never in contact with the soybean root environment before, an acclimation step of these bacteria to the soybean rhizosphere and the *Bradyrhizobium* partners was undergone before initiating the SynCom selection. To this end, each plant was inoculated with 1 mL bacterial culture containing the mixture of 14 rhizosphere isolates and the 9 *Bradyrhizobium* spp. strains.

Plants were maintained under controlled conditions in a greenhouse at La Plata, Province of Buenos Aires, Argentina (34°90′75.2″ S; 57°94′18.9″ W) at 28°C/22°C (day/night) with artificial light supplementation to complete 16 h photophase and watered with sterile distilled water as required. At 15 days after planting (DAP), an additional watering with sterile MFS was supplied. After 30 DAP, plants were removed, the potting material was poured into a sterilized container, the stems were cut off, and the vermiculite was carefully loosened from the roots using a sterile metal spatula. The roots were then placed in a 500 mL Erlenmeyer flask containing 250 mL MFS and shaken for 2 h to dislodge the rhizosphere community. The roots were discarded, and the vermiculite was separated by low-speed centrifugation (3,000 × *g,* 5 min). Rhizosphere bacteria were collectively recovered in the supernatant and either seeded directly into G2MY and cultured at 28°C and 180 rpm for 48 h or stored at −80°C for DNA extraction.

For the next rounds of selection, replicate cultures with different strain compositions were required. To prepare such replicas in a short time, the dilution-to-extinction strategy was employed (35). Serial dilutions were plated on solid G2MY (1.5% agar) until no colonies were observed from a 100-μL sample. The dilution previous to that was registered as extinction dilution and employed for all the successive inoculation rounds. One-milliliter aliquots of this dilution (~50–100 bacteria mL^−1^) were reinoculated in 10 replicated flasks containing 10 mL G2MY in order to favor the growth of mixtures with different compositions in each replica and grown to OD_500_ ~0.5.

In parallel, all nodules were detached from the roots and surface-sterilized as described (2). Then, nodules were crushed, and aliquots of rhizobia from each nodule were either individually stored at −80°C to obtain *Bradyrhizobium* DNA or mixed and divided into 10 aliquots to inoculate the 10 G2MY broths containing the rhizosphere bacteria mixture. In this way, each flask, which contained a sub-population of rhizosphere bacteria and the totality of nodule bacteria, was used to inoculate five soybean plants. Therefore, we expected that each five-plant set would receive a given composition of rhizosphere bacteria, while the *Bradyrhizobium* spp. were forced to compete for soybean nodulation. As a control, five plants were inoculated with sterile G2MY and, as a reference, the strain *B. japonicum* E109 grown in G2MY was inoculated in another five plants. After inoculation, all the plants were grown in the greenhouse as before. At 30 DAP, leaf chlorophyll contents were measured on the upper fully expanded trifoliate leaf of each plant with a portable chlorophyllometer and expressed in SPAD units, which represent leaf greenness (37), as an indirect measure of N_2_ fixation performance (2). The three plants with the highest SPAD values were chosen for the next round of selection and processed as before to obtain rhizosphere and nodule bacteria. Samples were split into two parts; one was kept at −80°C, and the other was processed for inoculation as before. The entire procedure was repeated eight times between April and December 2024.

### DNA extraction and amplicon sequencing

Genomic DNA was obtained from samples stored at –80°C. DNA integrity was evaluated by agarose gel electrophoresis and quantified using a NanoDrop 1000 spectrophotometer (Thermo Fisher Scientific) or a Qubit 3.0 fluorometer (Invitrogen). The bacterial isolates used to assemble the initial inoculum were taxonomically classified based on full-length 16S rRNA gene sequencing (V1–V9 region), which were obtained by PCR with the primers fd1 and rd1 (63).

For community analysis of inoculated rhizosphere samples after iterative passages, the hypervariable V3–V4 region of the 16S rRNA gene was amplified using primers Bakt_341F and Bakt_805R (64). Sequencing was performed using paired-end reads (2 × 300 bp) on an Illumina MiSeq platform at Macrogen Inc. (Korea). PCR conditions and primers followed protocols previously validated for both DNA fingerprinting and 16S amplification (3). Amplification of the *nosZ* gene was performed using primers nosZf and nosZr (43) under PCR conditions previously validated by Itakura et al. (65). For DNA sequencing of R_0_, R_A_, and HR*_i_* communities, total genomic DNA was extracted from root-adhered vermiculite samples using the QIAamp DNA Micro Kit (QIAGEN, the Netherlands), following the manufacturer’s instructions. Raw sequencing data have been deposited in the NCBI Sequence Read Archive under BioProject PRJNA1310012.

In addition, genomic DNA from *Bradyrhizobium* strains recovered from nodules post-infection was isolated, and DNA fingerprints were obtained using the BOX-A1R primer (53) under previously described PCR and electrophoresis conditions (2, 3). The resulting banding patterns were analyzed with GelCompare II v4.0 software (Applied Maths, Kortrijk, Belgium), considering a 1.5% optimization and 1.7% tolerance. Clustering of profiles was performed using the unweighted pair-group method with arithmetic mean (66), and branch distances were evaluated using the Jaccard coefficient (67).

### Data processing

For the taxonomic identification of the bacterial isolates used to assemble the initial inoculum, near full-length 16S rRNA gene sequences were analyzed with DNASTAR Lasergene (DNAStar, Inc.), compared against GenBank databases via NCBI BLAST, and aligned using MUSCLE. Phylogenetic trees were constructed in MEGA 11 using the neighbor-joining method (68). For the tree in Fig. 1, the Kimura 2-parameter model with gamma distribution (K2+G) was selected as the best-fit model, and node support was assessed with 1,000 bootstrap replicates (69, 70). The models used for trees in Fig. S6 are explained there.

For community-level analysis of rhizospheric samples obtained during the recurrent inoculation experiment, raw V3–V4 amplicon sequences were processed in the Mothur package (Galaxy Version 24.2.1.0) following the standard operating procedure (71). Reads were quality-checked and trimmed using Trimmomatic (Galaxy Version 0.36.0) (72). Paired-end reads were merged with the MAKE.CONTIGS command, and those containing ambiguous bases or homopolymers longer than eight bases were discarded. Sequences were aligned to the SILVA v.132 reference database (73), and poorly aligned reads were removed. Denoising was performed with the PRE.CLUSTER command (allowing ≤2 nucleotides), and chimeras were filtered using CHIMERA.VSEARCH. Remaining sequences were taxonomically classified with CLASSIFY.SEQ, and reads identified as chloroplasts or mitochondria were removed. Sequences were clustered into OTUs at 97% similarity (CLUSTER.SPLIT, taxonomic level 4, cut-off 0.03), and representative sequences were assigned taxonomy with CLASSIFY.OTU. Alpha-diversity indices (Sobs, Chao1, Shannon, inverse Simpson) were calculated using SUMMARY.SINGLE. Beta diversity was assessed descriptively using Bray-Curtis dissimilarity matrices (DIST.SHARED command), and community structure was visualized through NMDS.

### Statistical analysis

Chlorophyll contents were analyzed using one-way analysis of variance, followed by Tukey’s test (*P* < 0.05), as previously described (2). Diversity metrics and ordination plots for microbial communities were generated using Mothur (71) and visualized in R.

## Data Availability

Supplementary material may be found at http://sedici.unlp.edu.ar/handle/10915/191256.
